# Preharvest Treatment with 24-Epibrassinolide Enhances Resilience to Fruit Cracking, Yield and Quality Traits in Two Sweet Cherry Cultivars

**DOI:** 10.3390/ijms27062793

**Published:** 2026-03-19

**Authors:** Fernando Garrido-Auñón, Jenifer Puente-Moreno, María Emma García-Pastor, Vicente Agulló, Daniel Valero, María Serrano

**Affiliations:** 1 Department of AgroFood Technology, Escuela Politécnica Superior de Orihuela (EPSO), Instituto de Investigación e Innovación Agroalimentario y Agroambiental (CIAGRO), University Miguel Hernández, Ctr. Beniel km. 3.2, 03312 Orihuela, Alicante, Spain; fgarrido@umh.es (F.G.-A.); vagullo@umh.es (V.A.); daniel.valero@umh.es (D.V.); 2 Department of Applied Biology, Escuela Politécnica Superior de Orihuela (EPSO), Instituto de Investigación e Innovación Agroalimentario y Agroambiental (CIAGRO), University Miguel Hernández, Ctra. Beniel km. 3.2, 03312 Orihuela, Alicante, Spain; jpuente@umh.es (J.P.-M.); m.garciap@umh.es (M.E.G.-P.)

**Keywords:** anthocyanins, colour, firmness, *Prunus avium* L., ‘Skeena’, ‘Sunburst’

## Abstract

Sweet cherry (*Prunus avium* L.) is a highly appreciated fruit species for consumption but susceptible to climate change-induced weather, such as heavy rainfall, which catastrophically compromises yield and commercial fruit quality. Brassinosteroids (BRs) represent a novel biologically safe class of hormones that have been shown to increase plant resilience against these adversities and enhance crop yield and fruit quality in some fruit species. The main aim of this study was to evaluate the potential efficacy of the preharvest foliar spray treatments with 24-epibrassinolide (24-BL) at 0.01, 0.1 and 1 µM on crop yield, cracking incidence and fruit quality of ‘Sunburst’ and ‘Skeena’ sweet cherry cultivars, during two seasons with different weather conditions (2022 and 2023). Results revealed that 24-BL treatments improved fruit growth, fruit weight, and increased commercial crop yield, especially at 0.1 µM during the first season. Notably, in 2023, when extreme rainfall occurred, 24-BL at 0.01 and 0.1 µM significantly decreased cracking incidence by up to 50% for ‘Skeena’. Additionally, firmness, red colour and bioactive compounds, such as total phenolics and total anthocyanins, were also found at higher levels in fruits from 24-BL-treated trees compared to controls, in both cultivars and years. In conclusion, the foliar spray application of 24-BL at 0.01 µM and, especially at 0.1 µM, can be a useful and eco-friendly tool to reduce cracking incidence, improve crop yield and enhance sweet cherry quality traits regardless of environmental negative events, such as heavy rainfall. Importantly, the enhancement of bioactive compounds would promote additional antioxidant properties and enhance health benefits to consumers.

## 1. Introduction

Sweet cherry (*Prunus avium* L.) is a highly demanded fruit which belongs to the Rosaceae family. Last official data estimated the total sweet cherry production in Spain during 2023 season at 104,470 tons, which represented a reduction of 11,600 tons, losing two positions as one of the main crop producer countries around the world, with respect to 2022 [1].

‘Sunburst’ and ‘Skeena’ are two late-season sweet cherry cultivars which are normally harvested at the end of the season in Spain (between June and July). On the one hand, ‘Sunburst’ presents a very big fruit size, a squashed form, deep red colour, medium firmness and sweet flavour, while ‘Skeena’ shows a gross size, a rounded form, maroon colour, high firmness and very good taste [2]. On the other hand, ‘Skeena’ has higher anthocyanin content than ‘Sunburst’, increasing its red coloration and phytochemical profile [3,4]. The phytochemical profile in sweet cherries is determined by bioactive compounds, mainly anthocyanins hydroxycinnamic acids and other phenolic compounds, which are found in different concentrations depending on cultivar, rootstocks and agricultural practices [5] and have been associated with the reduction and prevention of some human diseases such as inflammatory diseases, neuronal diseases or even cancer [6,7,8]. In addition, firmness and sweetness are very important organoleptic parameters appreciated by consumers in sweet cherries, which show important differences among cultivars and are also influenced by agronomical and environmental factors, although in general, firmness decreases with the ripening process while total soluble solid content increases [9,10].

According to the European Commission, high temperatures, as well as more frequent extreme rainfalls, will affect Europe in next years because of climate change [11]. These conditions are happening nowadays, and an abnormally high rainfall occurred in the Southeast of Spain in 2023 during the month of May [12], which catastrophically reduced national sweet cherry production due to fruit cracking. Fruit cracking is one of the main economic issues in sweet cherry fruits, leading to huge reductions in growers’ income [13], although susceptibility to cracking depends on cultivar cuticular characteristics [14]. Additionally, high temperatures can negatively affect sweet cherry quality, producing effects such as dried fruit stems or a sunken epidermis [15]. Thus, the productivity and quality of crops are strongly shaped by climatic and environmental conditions such as temperature fluctuations or irregular precipitation, which influence overall crop performance and fruit quality from preharvest to postharvest. Apart from reducing crop yield and fruit quality, failures in dormancy break have been observed in *Prunus* species, namely peach, plum, almond, apricot and sweet cherry, which are shifting the geographic distribution of sustainable areas for crop cultivation [16,17].

Recent research has focused on finding effective tools to improve crop resilience against climatic adverse events under the present climate change scenario, with additional effects on fruit quality at harvest. Elicitors are molecules that activate a plant’s biochemical mechanisms, leading to enhanced plant resilience against stressors [18]. New elicitors such as melatonin [19], gibberellic acid [20], salicylic acid and acetyl salicylic acid [21], have been applied as preharvest treatments to improve the commercial quality and enhance antioxidant compounds of different sweet cherry cultivars. Apart from that, other preharvest strategies such as new irrigation systems [22], biofilm coatings [23] or arginine treatment [24] have been studied recently to improve sweet cherry quality. So, the research of new compounds, which are safer and more eco-friendly than traditional phytochemicals, is being carried out to avoid pre and postharvest economical losses in terms of environmental perspectives and fruit quality.

In this context, brassinosteroids (BRs) are presented as new potential molecules to increase plant resilience. The brassinosteroids family is a group of plant steroid hormones which is defined as the 3-oxygenated (20β)-5α-cholestane-22α,23α-diols, or their derived compounds, bearing additional alkyl or oxy substituents [25]. Nowadays, BRs are the sixth class of phytohormones, and present important roles in cell division, elongation, and expansion [26]. In addition, some reports have claimed the potential effect of BRs to counter abnormal problems caused by climate change, such as flooding, drought or heat, and biotic stresses (virus, bacteria or fungi), since BRs pre and postharvest treatments have demonstrated positive effects on yield, growth, ripening and quality of different fleshy fruits [18,27]. Indeed, the preharvest use of the BR analogue BIOBRAS-16 at 0.1 mg L^−1^ has demonstrated increases on the yield of yellow passion fruit plants [28]. Furthermore, preharvest treatments with 24-epibrassinolide (24-BL), which is the main brassinolide used in physiological studies and a biologically safe molecule for consumers’ health [18,29], has revealed improvements on crop yield and fruit quality at harvest for several red fruits, such as blood oranges [30], strawberries [31], and grapes [32], increasing important commercial parameters as anthocyanin content and, thus, red coloration in an eco-friendly way. Apart from that, other preharvest studies on nutmegs [33] and sweet corn [34] have also claimed improvements in both yield and commercial quality parameters. According to the postharvest studies, there was one work where 24-BL was applied in postharvest at 5 µM on sweet cherries cv. ‘Mei Zao’ leading to improve firmness due to a suppression of the expression of genes codifying the enzymes involved in the pectin degradation under ‘ultra-low’ cold storage [35], and another where the postharvest application of BRs at 2 µM reduced the chilling injury incidence on sweet cherries cv. ‘Tieton’ [36]. Nevertheless, according to the revised literature there are no reports about the preharvest use of 24-BL to enhance yield, quality and bioactive compounds at harvest in sweet cherry.

Therefore, the main objective of this study was to evaluate for the first time the effects of preharvest treatment with 24-BL (applied in a range of micro molar concentration) on cracking incidence, yield and fruit quality parameters at harvest in ‘Sunburst’ and ‘Skeena’ cultivars, during the 2022 and 2023 seasons. This study presents the preharvest treatment with 24-BL as a potential, innovative, safe and eco-friendly tool to improve crop yield and quality parameters in two sweet cherry cultivars. This strategy would reduce food waste, economical losses and enhance bioactive compound, improving the quality of the sweet cherry cultivars ‘Sunburst’ and ‘Skeena’ at harvest.

## 2. Results

### 2.1. Effects of 24-BL Treatments on Fruit Growth and Cracking Incidence

**Figure 1:**
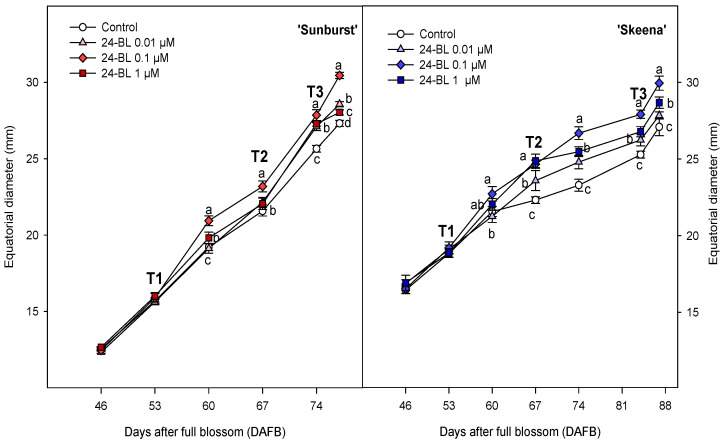
Equatorial diameter (mm) of ‘Sunburst’ and ‘Skeena’ sweet cherry fruits for control and preharvest treated fruits with 24-epibrassinolide (24-BL) at 0.01, 0.1 and 1 µM in 2022. T1, T2 and T3 are the treatment dates. Data are the mean ± SD. Different letters show significant differences (*p* < 0.05) among treatments for each day after full blossom (DAFB) and cultivar.

Fruit growth was measured in both sweet cherry cultivars from full blossom till harvest every week, three days before harvest and at harvest (Figure 1). Regarding the ‘Sunburst’ cultivar, a significant (*p* < 0.05) increase of ~9% on the equatorial diameter in 0.1 µM treated fruits compared to control at 60 days after full blossom (DAFB) was observed. Finally, after 77 DAFB, all the 24-BL treated fruits showed significant (*p* < 0.05) higher values than the controls, with 0.1 µM the treatment leading to the highest diameter (30.45 ± 0.21 mm). For the ‘Skeena’ cultivar, 24-BL at 0.1 µM increased significantly (*p* < 0.05), by 15%, the sweet cherry equatorial diameter with respect to the control at 67 DAFB and reached the significant (*p* < 0.05) highest value at 87 DAFB (29.95 ± 0.46 mm). Lastly, lower but significant (*p* < 0.05) increases were observed for 24-BL at 0.01 µM and 1 µM, with respect to the control, after T2 and T3 24-BL applications.

**Figure 2:**
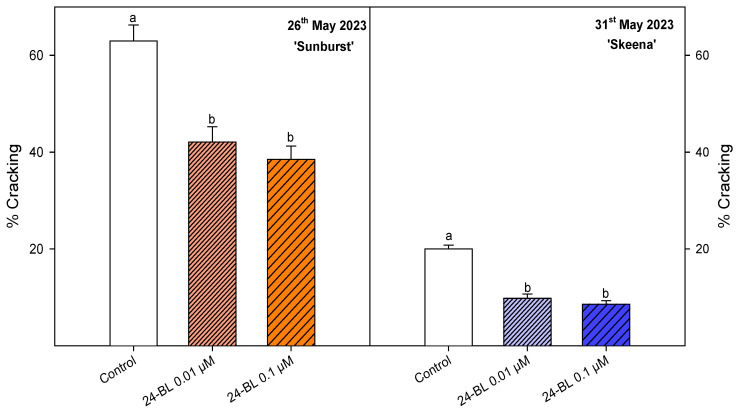
Percentage of fruits (%) that suffered cracking in 2023 in ‘Sunburst’ and ‘Skeena’ cultivars as affected by preharvest treatments fruits with 24-epibrassinolide (24-BL) at 0.01 and 0.1 µM. Data are the mean ± SD. Different letters show significant differences (*p* < 0.05) among treatments for each cultivar.

As a consequence of the extreme rainfall that affected the Southeast of Spain in May of 2023, a high incidence of cracking was observed, affecting to more than 60% of fruits in control trees for ‘Sunburst’ and ca. 20% in ‘Skeena’. However, 24-BL at 0.01 and 0.1 µM significantly (*p* < 0.05) decreased the incidence of cracking on ‘Sunburst’ and ‘Skeena’, by more than 30% and 50%, respectively, with respect to the control (Figure 2).

### 2.2. Sweet Cherry Crop Yield and Fruit Weight

**Figure 3:**
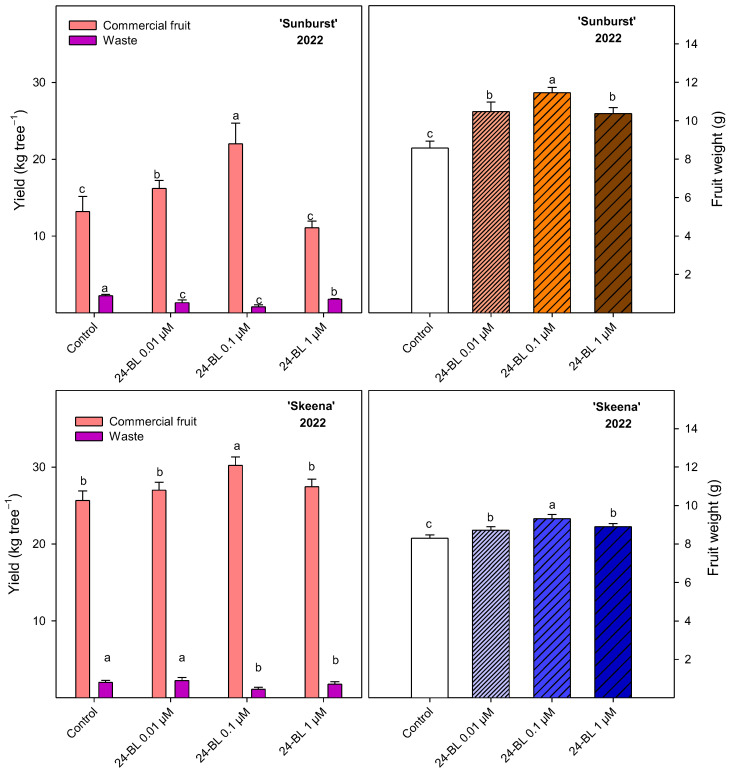
Yield (kg tree^−1^) of commercial and non-commercial fruit (waste) and fruit weight (g) in ‘Sunburst’ and ‘Skeena’, for control and preharvest treated sweet cherries with 24-epibrassinolide (24-BL) at 0.01, 0.1 and 1 µM in 2022 season. Data are the mean ± SD. Different letters show significant differences (*p* < 0.05) among treatments for each cultivar.

Crop yield and fruit weight at harvest were calculated for both cultivars during the first season (Figure 3). For the ‘Sunburst’ cultivar, firstly 24-BL at 0.1 µM (22.01 ± 2.70 kg tree^−1^) and secondly 24-BL at 0.01 µM (16.21 ± 1.06 kg tree^−1^) showed the significant (*p* < 0.05) highest values of crop commercial yield, with respect to the other treatments. Additionally, all treatments increased the fruit weight between 20 and 30% with respect to the control and significantly reduced (*p* < 0.05) the number of fruits that were designated to waste. For the ‘Skeena’ cultivar, 24-BL at 0.1 µM was the only treatment that significantly (*p* < 0.05) increased both crop yield (30.22 ± 1.10 kg tree^−1^) and fruit weight (9.31 ± 0.21 g) with respect to control, though 24-BL at 0.01 µM and at 1 µM also significantly (*p* < 0.05) increased fruit weight. In addition, 24-BL at 0.1 and at 1 µM significantly reduced (*p* < 0.05) the wasted fruits.

### 2.3. Sweet Cherry Quality Parameters

**Figure 4:**
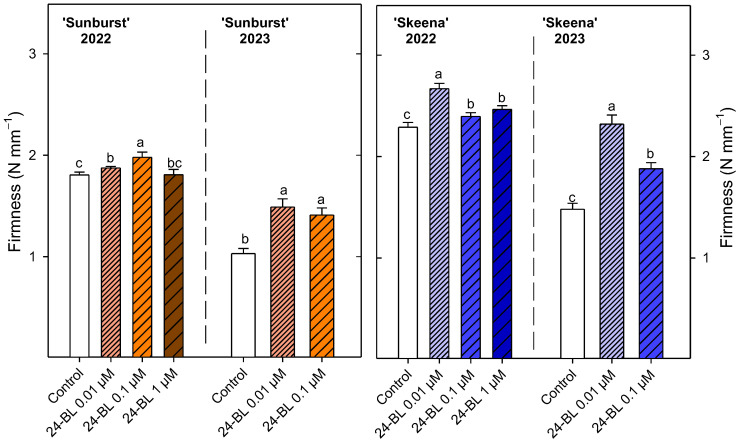
Firmness (N mm^−1^) at harvest in ‘Sunburst’ and ‘Skeena’ sweet cherries from control and trees treated preharvest with 24-epibrassinolide (24-BL) at 0.01, 0.1 and 1 µM in 2022, and at 0.01 and 0.1 µM in 2023. Data are the mean ± SD. Different letters show significant differences (*p* < 0.05) among treatments for each cultivar and season.

With respect to the 2022 season, fruit of the ‘Sunburst’ cultivar showed the significantly (*p* < 0.05) highest firmness (Figure 4) values after being treated with 24-BL at 0.1 µM (1.98 ± 0.05 N mm^−1^). In the ‘Skeena’ cultivar, fruit firmness was significantly increased (*p* < 0.05) for all treatments with respect to controls, 24-BL at 0.01 µM being the treatment which showed the highest significant (*p* < 0.05) value (2.67 ± 0.05 N mm^−1^). In relation to 2023, the firmness of fruits from control and treated trees was ~40% and ~30% lower than in 2022 for ‘Sunburst’ and ‘Skeena’, respectively. However, differences were maintained for both cultivars between treatments. Thus, in the ‘Sunburst’ cultivar sweet cherries from trees that were treated preharvest with 24-BL at 0.01 and at 0.1 µM showed significantly (*p* < 0.05) higher values than controls. Accordingly, 24-BL at 0.01 and at 0.1 µM significantly (*p* < 0.05) increased the firmness, by 57% and 27%, respectively, compared with the control in the ‘Skeena’ cultivar.

**Figure 5:**
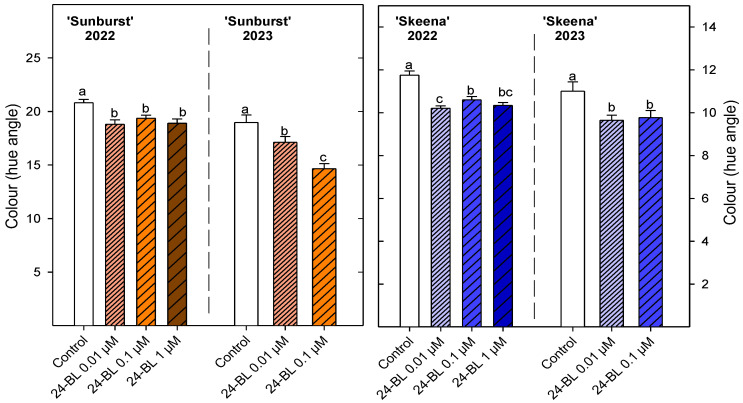
Colour (hue angle) at harvest in ‘Sunburst’ and ‘Skeena’ sweet cherries from control and trees treated preharvest with 24-epibrassinolide (24-BL) at 0.01, 0.1 and 1 µM in 2022, and at 0.01 and 0.1 µM in 2023. Data are the mean ± SD. Different letters show significant differences (*p* < 0.05) among treatments for each cultivar and season.

In both sweet cherry cultivars and years, all the fruits from trees treated preharvest with any concentration of 24-BL had significantly (*p* < 0.05) lower hue angle (°h) values than the control ‘Skeena’ (Figure 5). In 2023, hue angles values were lower than in 2022 in control and treated fruits, although differences between control and treated fruits were maintained. The treatment with 24-BL at 0.1 µM significantly reduced (*p* < 0.05) the hue angle, by 23%, with respect to the control for ‘Sunburst’ cultivar. In relation to the ‘Skeena’ cultivar, preharvest treatment with 24-BL at 0.01 and at 0.1 µM also showed lower values (9.64 ± 0.24 and 9.77 ± 0.33, respectively) compared to control (11.00 ± 0.43).

**Figure 6:**
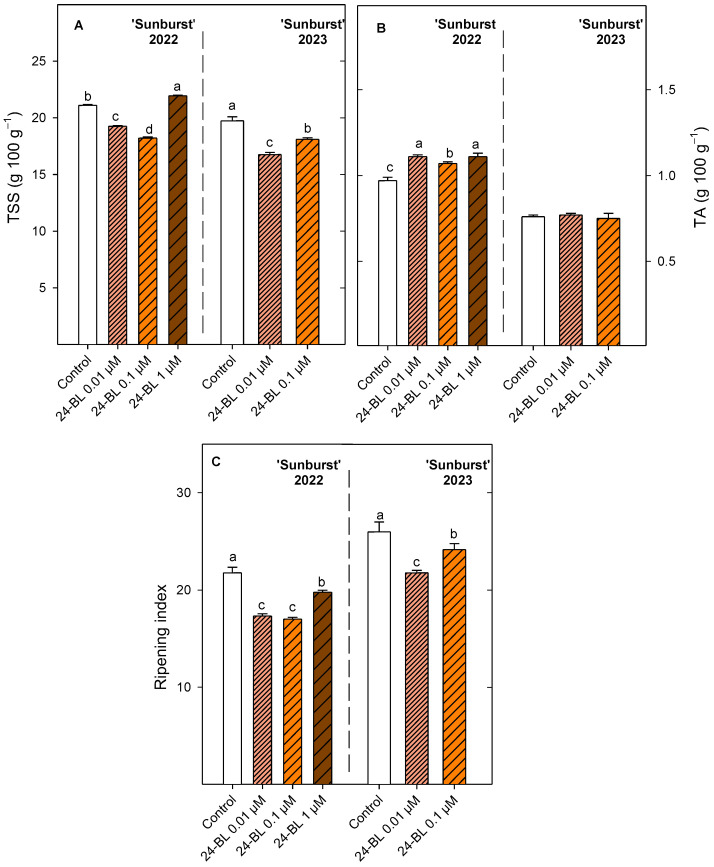
(**A**) Total soluble solids (TSS) (g 100 g^−1^), (**B**) total acidity (TA) (g 100 g^−1^) and (**C**) ripening index at harvest in ‘Sunburst’ fruit from control and preharvest treated trees with 24-epibrassinolide (24-BL) at 0.01, 0.1 and 1 µM in 2022, and at 0.01 and 0.1 µM in 2023. Data are the mean ± SD. Different letters show significant differences (*p* < 0.05) among treatments for each cultivar and season.

For total soluble solids (TSS) in ‘Sunburst’, 24-BL at 0.1 µM was the treatment which led to the significant (*p* < 0.05) lowest values, followed by 0.01 µM concentration, in 2022. In addition, in the 2023 season, 24-BL at 0.01 µM and 0.1 µM also led to significantly (*p* < 0.05) lower values than the control (Figure 6). On the contrary, all 24-BL treatments significantly increased (*p* < 0.05) total acidity (TA) with respect to the control (0.97 ± 0.02 g 100 g^−1^) in the 2022 season in the ‘Sunburst’ cultivar (Figure 6). In the 2023 season, TA did not show significant (*p* ≥ 0.05) differences between treatments in ‘Sunburst’. The ripening index (TTS/TA) was significantly (*p* < 0.05) lower (Figure 6) in 24-BL treatments than in the control for both seasons, though the ripening index was lower in the first season than in the second one.

**Figure 7:**
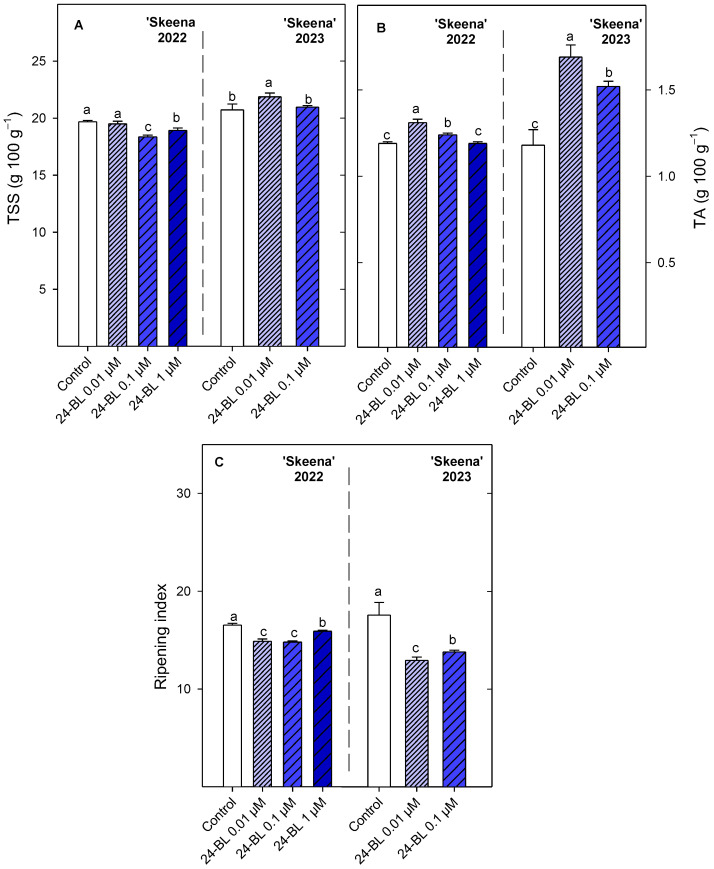
(**A**) Total soluble solids (TSS) (g 100 g^−1^), (**B**) total acidity (TA) (g 100 g^−1^) and (**C**) ripening index at harvest in ‘Skeena’ fruit from control and preharvest treated trees with 24-epibrassinolide (24-BL) at 0.01, 0.1 and 1 µM in 2022, and at 0.01 and 0.1 µM in 2023. Data are the mean ± SD. Different letters show significant differences (*p* < 0.05) among treatments for each cultivar and season.

For ‘Skeena’ in the 2022 season, the significant (*p* < 0.05) lowest TSS values (18.36 ± 0.15 g 100 g^−1^) were observed after the treatment with 24-BL at 0.1 µM (Figure 7). During the following season, 24-BL at 0.01 µM obtained the significant (*p* < 0.05) highest values (21.87 ± 0.33 g 100 g^−1^). In relation to TA, 24-BL 0.01 µM showed the highest (1.31 ± 0.02 g 100 g^−1^) significant (*p* < 0.05) values compared with all the treatments in the 2022 season. Similarly, firstly, 24-BL at 0.01 µM and secondly 24-BL at 0.1 µM significantly increased (*p* < 0.05) the TA, by 40% and 30%, in relation to the control during the 2023 season. According to these values, all 24-BL treatments significantly (*p* < 0.05) reduced the ripening index (Figure 7) with respect to the control in 2022 and 2023, and 24-BL at 0.01 µM was the treatment with the significant (*p* < 0.05) lowest RI value (12.94 ± 0.34), followed by 24-BL at 0.1 µM (13.80 ± 0.19) in 2023.

### 2.4. Anthocyanins and Phenols on Sweet Cherry Flesh

**Figure 8:**
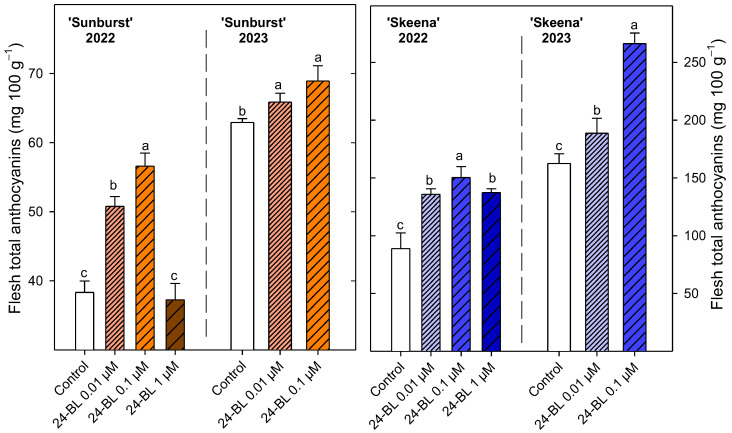
Total anthocyanin content (mg 100 g^−1^) at harvest in ‘Sunburst’ and ‘Skeena’ fruit from control and trees treated preharvest with 24-epibrassinolide (24-BL) at 0.01, 0.1 and 1 µM in 2022 and at 0.01 and 0.1 µM in 2023. Data are the mean ± SD. Different letters show significant differences (*p* < 0.05) among treatments for each cultivar and season.

For the ‘Sunburst’ cultivar in the first season, 24-BL at 0.1 µM showed the highest significant (*p* < 0.05) total anthocyanin values (56.6 ± 1.88 mg 100 g^−1^) (Figure 8); after that, 24-BL at 0.01 µM also significantly increased (*p* < 0.05) the total anthocyanin content (50.78 ± 1.43 mg 100 g^−1^) compared with the other treatments. Similar results were obtained during the second season, since 24-BL at 0.01 and at 0.1 µM significantly (*p* < 0.05) increased the total anthocyanin content compared with the control. Regarding the ‘Skeena’ cultivar, all 24-BL significantly increased (*p* < 0.05) the total anthocyanins in the first season, being 24-BL at 0.1 µM which showed the highest significant (*p* < 0.05) increase of 69% (150.23 ± 9.49 mg 100 g^−1^) with respect to the control. Similar results were obtained in the second season in the ‘Skeena’ cultivar, since 24-BL at 0.1 µM led to a significant 64% (*p* < 0.05) increase in the anthocyanin content with respect to the control.

**Figure 9:**
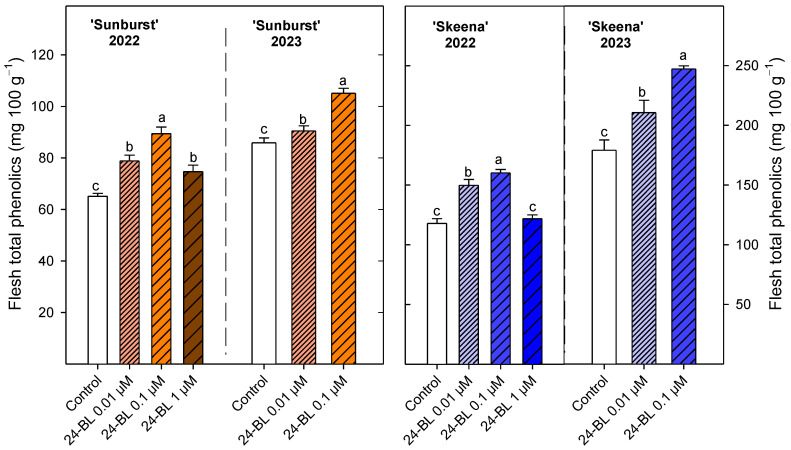
Total phenolic content (mg 100 g^−1^) at harvest in ‘Sunburst’ and ‘Skeena’ fruit from control and trees treated preharvest with 24-epibrassinolide (24-BL) at 0.01, 0.1 and 1 µM in 2022 season, and at 0.01 and 0.1 µM in 2023 season. Data are the mean ± SD. Different letters show significant differences (*p* < 0.05) among treatments for each cultivar and season.

In general, all 24-BL treatments significantly increased (*p* < 0.05) the total phenolic content (Figure 9) in both cultivars and growing seasons. The highest significant (*p* < 0.05) values in ‘Sunburst’ were shown by 24-BL at 0.1 µM (89.44 ± 2.55 mg 100 g^−1^) in the 2022 and 2023 seasons. With respect to the ‘Skeena’ cultivar, in 2022, 24-BL at 0.1 showed the highest (160.14 ± 2.89 mg 100 g^−1^) significant (*p* < 0.05) values, followed by 24-BL at 0.01 µM (149.77 ± 4.93 mg 100 g^−1^). Additionally, in the second year, firstly 24-BL at 0.1 µM (247.21 ± 2.61 mg 100 g^−1^) and secondly 24-BL at 0.01 µM (210.65 ± 10.26 mg 100 g^−1^) showed significant (*p* < 0.05) higher total phenolic content than the control (179.16 ± 8.57 mg 100 g^−1^).

## 3. Discussion

Climate change is currently recognised as a major global challenge that has caused an increase in the Earth’s average temperature of 1.3 °C in 2024 [37]. The increased frequency and intensity of extreme climatic events, including heavy rainfall, droughts, and heat waves, have become a major threat to fruit production systems, negatively affecting crop yield and fruit quality, and resulting in significant economic losses. Consequently, recent research is focused on the development of agronomic strategies aimed at enhancing crop resilience, productivity, and fruit quality under adverse climatic scenarios. In this context, 24-BL emerges as a promising eco-friendly plant growth regulator capable of enhancing fruit quality and stress tolerance [25,26,38].

The sweet cherry crop is highly susceptible to rain-induced cracking, with certain cultivars, such as ‘Skeena’, being particularly sensitive to this problem [14]. Cracking is a physiological disorder that affects the cuticle of sweet cherries during their growth, and increases in extreme rain and high humidity conditions, being one of the most severe quality defects in sweet cherries [39]. In the present study, the 2023 growing season was characterised by exceptionally high precipitation, with May registering the highest accumulated rainfall recorded in the Alcoy area (Alicante, Spain) since 1950 (156 L m^−2^), severely compromising commercial sweet cherry yield [12]. This extreme scenario provided a suitable framework to evaluate the effectiveness of 24-BL to mitigate cracking and preserve fruit quality.

Results demonstrated that preharvest applications of 24-BL at 0.01 and 0.1 µM significantly reduced cracking incidence in both ‘Sunburst’ and ‘Skeena’ cultivars (Figure 2), highlighting its protective role under extreme rainfall conditions. Therefore, the decrease in cracking incidence observed after 24-BL treatments represents a major improvement in sweet cherry quality under adverse climatic conditions. Although the effect of preharvest treatment with BRs on cracking reduction on sweet cherries has not been previously reported, postharvest studies have reported similar protective effects [36]. Zhu et al. observed that 2 μM BRs significantly reduced postharvest-induced cracking by 25.34% after 28 days of cold storage in ‘Tieton’ cherries, associated with the BRs effect on the reduction in the cell wall-modifying enzymes and maintenance of firmness [36]. Additionally, BRs-treated fruits accumulated higher levels of antioxidant compounds, such as phenols and flavonoids, which could inactivate reactive oxygen species and alleviate the symptoms of sweet cherry injury. Notably, as in the present study, lower BRs concentrations exhibited stronger protective effects, highlighting the relevance of optimising dose selection [36].

In addition to this positive effect, 24-BL has been connected with different roles in multiple physiological processes regulation, including cell division, cell expansion, and vascular differentiation [26]. The present results revealed that preharvest treatment with 24-BL significantly increased equatorial fruit diameter throughout sweet cherry development, particularly at concentrations of 0.01 µM and 0.1 µM for ‘Sunburst’ and ‘Skeena’, respectively (Figure 1). BRs have been widely reported to regulate fruit and grain size through molecular signalling networks. In rice, the gene *SMALL GRAIN 2* (SG2) encodes a plant specific protein in response to BRs signalling, which regulates grain size by interacting with the *Oryza sativa* OVATE family protein 19 (OsOFP19) [40]. Consistently, preharvest BRs treatments increased fruit size and weight in grapes, strawberries, mangoes, persimmons, sugar apples and giant pumpkin due to improvements in the carbon dioxide (CO_2_) assimilation levels [41,42,43,44,45,46]. Moreover, studies using BRs-insensitive tomato mutants exhibited an impaired expression of fruit-specific genes, such as sucrose transporters, protein kinases, and auxin-responsive transcription factors, confirming the direct involvement of BRs in fruit development [47].

Furthermore, the results reported an increase in fruit weight after preharvest BRs treatments in both cultivars, with the highest effects found for 0.1 µM, leading to significant increases in commercial crop yield (Figure 3). This increase may be related to enhanced photosynthetic capacity and carbon assimilation efficiency induced by BRs signalling. In fact, previous studies in cucumber demonstrated that BRs application increased CO_2_ assimilation, chlorophyll content, leaf area, and photosynthetic enzyme activities [48]. Additionally, crosstalk between gibberellic acid and BRs in regulating cell elongation and growth has been described in *Arabidopsis* [49]. Nevertheless, it is worth noting that the effect of 24-BL in increasing crop yield was dependent on the applied dose, the most effective being 0.1 µM for both cultivars. However, treatment with 24-BL at 1 µM concentration did not show significant differences in crop yield for either Sunburst or Skeena compared with the control, although it increased fruit weight (Figure 3). These results are in accordance with previous research on blood oranges in which 0.1 µM 24-BL treatment increased crop yield but no additional effect was observed for 1 µM [30]. Furthermore, the increase in the number of harvested fruits has also been described in other BRs treated species such as mangoes, passion fruit, apricots or Navel oranges, which was related with an increase in fruit set and a delay in fruit abscission, contributing to overall yield improvement, although the most effective dose was different depending on cultivar [48,50]. Moreover, these effects of 24-BL may vary depending on application times, cultural practices and environmental conditions, as well as the studied fruit species or cultivar [18].

Firmness and ripening index are key parameters determining sweet cherry consumer acceptance and can vary depending on agricultural and environmental factors. Indeed, sweet cherry firmness was lower in the 2023 season than in 2022 probably due to the extreme rainfall. Independently of that, the preharvest 24-BL treatment increased fruit firmness in both cultivars and seasons (Figure 4) and decreased the ripening index (Figure 6 and Figure 7). These results are consistent with previous reports in blood orange, strawberry and sugar apple crops, where preharvest BRs treatments increased firmness at harvest and potentially extended postharvest shelf life [30,42,51]. The firmness enhancement in sweet cherries is mainly attributed to BRs-mediated inhibition of pectin modifying enzymes such as polygalacturonase (PG), pectin methylesterase (PME), and pectate lyase (PL), since the postharvest BRs treatment reduced water-soluble and ionically soluble pectin levels [36]. Accordingly, in peaches, BRs at a concentration of 10 µM maintained cell wall integrity by inhibiting the expression of genes *PpPME1/3*, *PpPG*, *PpARF2*, and *PpGAL2/16*, related with pectin degradation, leading to a delay in the softening process [52]. The present results show an improvement in firmness together with a reduction in the ripening index, as has been previously reported in blood oranges [30]. On the contrary, firmness retention after BRs treatment was not concurrent with lower ripening index during storage in strawberries and persimmon fruits [31,44].

In addition to firmness and ripening index, colour represents another important sweet cherry quality parameter. Although ‘Sunburst’ and ‘Skeena’ cultivars are different in colour intensity, ‘Sunburst’ being described as having a deep red colour and ‘Skeena’ a maroon colour [2], the preharvest treatment with 24-BL reduced the hue angle showing an increased red colouration in both cultivars and seasons (Figure 5). Previously, postharvest BRs treatments in ‘Tieton’ sweet cherries increased a* colour parameter, which is positively related to the red colour in the CIE L* a* b* space [36]. This effect can be associated with a marked increase in total anthocyanin content. Although, there are no reports where anthocyanins have been analysed in sweet cherries after the BRs treatment, other elicitors, such as melatonin, have been previously described to improve anthocyanin content and then red colouration in ‘Sunburst’ [53]. In addition, some reports have claimed increases in the total anthocyanin content after preharvest or postharvest BRs treatments in strawberries, grapes, persimmons, and mangoes [32,43,44,51]. This effect is consistent with the present results (Figure 8), confirming the stimulatory role of BRs on pigment biosynthesis. The enhanced accumulation of anthocyanins after the BRs treatment is likely mediated by activation of the phenylpropanoid pathway, particularly through increased activity of phenylalanine ammonia-lyase and chalcone isomerase [54]. Furthermore, BRs regulate transcription factors related with anthocyanin accumulation such as brassinazole-resistant 1 (BZR1) and production of anthocyanin pigment 1 (PAP1) in *Arabidopsis thaliana* [55]. Then, 24-BL treatment can effectively maintain the activity of the relevant enzymes that cause colour changes in fruits and vegetables [36]. Furthermore, BRs are known to have a cross-hormonal effect with other phytohormones such as auxins, gibberellins, and cytokinins, thereby modulating the ripening process and colour change [50]. Indeed, in grapes, which are non-climacteric fruits like sweet cherries, a greater increase in anthocyanin content was observed when gibberellins and BRs were applied together as compared to independent applications, suggesting that these hormones may have a crosstalk effect, as has also been observed in *Arabidopsis thaliana* [56,57].

In addition, 24-BL significantly increased total phenolic compounds (Figure 9), particularly at 0.01 and 0.1 µM. This effect has been previously reported in sweet cherries and other fruits following postharvest BRs treatments [18]. The enhanced phenolic content may be associated with the inhibition of phenolic-degrading enzymes such as polyphenol oxidase and peroxidase, as observed in peach and carambola fruit after postharvest BRs treatment [58,59]. However, this is the first report showing an increase in total phenolic and anthocyanin contents in sweet cherry due to BRs treatments during on-tree fruit development. Consequently, the accumulation of these bioactive compounds induced by 24-BL could substantially enhance the functional quality of sweet cherry fruit and derived juices, contributing to their antioxidant capacity and potential health-promoting properties [6,7,8]. Indeed, a recent in vivo study has claimed that dark sweet cherry anthocyanins can have a positive effect on breast cancer pulmonary metastasis [60].

In terms of cost-benefits of the 24-BL treatments, an increase in ca. 6 kg per tree was obtained with the best dose, 0.1 µM, which would lead to an increase of 12 euros per tree, according to the selling price for farmers in the 2024 season in Spain [61]. The cost of the 0.1 µM 24-BL treatment was ca. 13 euros, based on the price of the 24-BL used in this experiment (Sigma-Aldrich CAS Number: 72962-43-7). However, for practical purposes in commercial fields, not so pure 24-BL product could also be used and the cost of the treatment would be drastically reduced. Apart from that, it should also be noted that treated cherries were of higher quality and will likely be more accepted by consumers, which could also affect the final price.

## 4. Materials and Methods

### 4.1. Experimental Design, Cracking Incidence, and Evaluation of Crop Yield

The field experiments were carried out in a commercial crop located in Alcoy (Alicante, Spain, 38.649, −0.491) in sweet cherry trees (*Prunus avium* L.) of ‘Sunburst’ and ‘Skeena’ cv. during two different seasons: 2022 and 2023. Trees were treated with three different doses of 24-BL (0.01, 0.1 and 1 µM) during the first year (2022) and the best results were obtained for 0.01 and 0.1 µM doses, in terms on crop yield and commercial quality and no better effect were observed with the highest assayed dose. Thus, to optimise the range of 24-BL concentrations, the preharvest treatments were repeated with the lowest doses (0.01 and 0.1 µM) during the second year (2023). Sweet cherry trees were organised in 3 blocks of 3 trees (9 trees in total for each treatment). All sweet cherry trees were grown under similar agricultural practices and environmental conditions. Trees were foliar sprayed with the different 24-BL concentrations, containing 1 mL L^−1^ Elogium^®^ (Alquil-Poliglicol Ether 20%, *w*/*v*) as surfactant, by using a manual mist sprayer, (Agrodosmil Ganadera S.L., Villagonzalo Pedernales, Burgos, Spain) and control trees were sprayed in the same way with distilled water also containing 1 mL L^−1^ Elogium^®^. The foliar spray applications (1.5 L per tree) were performed at three key points of the sweet cherry development for both seasons: T1 at pit hardening (beginning of May), T2 at the beginning of colour changes (end of May–beginning June) and T3 three days before harvest. 24-BL was purchased from Sigma-Aldrich (Madrid, Spain, CAS Number: 72962-43-7) and dissolved in 15 mL of ethanol to prepare the different concentrations.

Sweet cherry growth was measured from full blossom till harvest every week, three days before harvest and at harvest. Equatorial diameters (mm) were measured in 30 sweet cherries for each tree. Fruits were harvested when they reached their commercial ripening stage, then fruit were separated into commercial and non-commercial and weighted to obtain commercial fruit and waste, respectively, as kg tree^−1^ according to commercial practices. In 2022, a random sample of 100 fruits per tree was collected to assess fruit weight. Only in the 2022 season could the sweet cherry crop yield be determined, since in May of 2023 extreme rainfall affected the Southeast of Spain, with an accumulated precipitation of 156 L m^−2^ in the local area of Alcoy making it the wettest month of May since 1950 in the province of Alicante [12], which catastrophically affected the crop yield. For that reason, in 2023 cracking incidence was quantitatively evaluated in the 24-BL-treated and control trees by counting the number of fruits showing cracking from a total of 50 fruits selected at random on one branch per each tree, with results were expressed as a percentage. Although crop yield could not be quantified in 2023, enough fruits were harvested to make analytical determinations. For both years and cultivars, a total of 150 homogenous fruits per treatment (50 fruits per replicate) were used to select three replicates of twenty fruits (*n* = 3) homogenous in size and colour and were transported immediately to the laboratory to conduct analytical determinations.

### 4.2. Fruit Quality Parameters

Colour, firmness, total soluble solids (TSS) and total acidity (TA) were determined as the main commercial quality parameters in sweet cherry. On the one hand, to measure sweet cherry colour, a Minolta colorimeter (CRC400, Minolta Camara Co., Tokyo, Japan) recorded L*, a* and b* coordinates in the CIE Lab systems. For each individual fruit, three readings were taken at three equidistant points of the equatorial fruit perimeter, and the hue angle (arctan b*/a*) was calculated to represent the red colour. On the other hand, fruit firmness was determined by using a TX-XT2i Texture Analyser (Stable Microsystems, Godalming, UK) equipped with a steel flat plate device, which applied a force to achieve a 3% deformation of the fruit equatorial diameter. Results were expressed as force-deformation ratio (N mm^−1^). For colour and firmness, data are the mean ± standard deviation (SD) of the three replicates each one composed of 20 fruits (*n* = 3).

Thereafter, sweet cherries of each replicate were cut and ca. 50 g were manually squeezed to measure TSS and TA. To analyse TSS a digital refractometer (model Atago PR-101, Atago Co., Ltd., Tokyo, Japan) was used. For each replicate and treatment, TSS was measured in duplicate at 25 °C, expressing the results (mean ± SD) as g 100 g^−1^. In a similar way, for each replicate, 1 mL of juice in 25 mL of distilled water was used to determine TA in duplicate by automatic titration (785 DMP Titrino; Metrohm, Herisau, Switzerland) with 0.1 N NaOH up to reaching pH 8.1, and results were expressed as the mean ± SD of g malic acid equivalent per 100 g^−1^ of fresh weight. Ripening index was calculated as the ratio TSS/TA. Finally, for each treatment and replicate, flesh was frozen at −20 °C until total anthocyanin and total phenolic content were measured.

### 4.3. Total Anthocyanin and Total Phenolic Content

To extract and measure total anthocyanin content in sweet cherry flesh, the protocol followed by Carrión-Antolí et al. [19] was adapted. Briefly, for each replicate 5 g of flesh were homogenised with 15 mL of methanol/hydrochloric acid/water (25:1:24, *v*/*v*/*v*), to extract total anthocyanins. The extract was centrifuged at 10,000× *g* for 10 min at 4 °C, and the supernatant was used to quantify total anthocyanins by reading absorbance at 520 nm. Total anthocyanin levels were expressed as mg 100 g^−1^ of cyanidin 3-glucoside equivalent (molecular weight 449.2 g mol^−1^ and molar absorption coefficient of 26,900 L cm^−1^). Similarly, to extract the total phenolic content 5 g of flesh were homogenised and extracted with 15 mL of water/methanol (2:8, *v*/*v*) containing 2 mM NaF, by using an Ultraturrax (T18, IKA, Berlin, Germany). The extracts were centrifuged for 10 min at 10,000× *g*. After the extraction, the colorimetric reaction was carried out adapting the method used by Díaz-Mula et al. [9]. For that, 200 μL of the supernatant were mixed with 500 μL of phosphate buffer (50 mM, pH 7.8) plus 2.5 mL of water-diluted Folin-Ciocalteau (Sigma-Aldrich, St. Louis, MO, USA) (1:10) reagent and the mix was incubated for 2.5 min at room temperature. Then, 2 mL of sodium carbonate (53 g L^−1^) were added and shaken vigorously. After that, the samples were incubated in a warm water bath at 50 °C for 5 min. Finally, the absorbance was measured at 760 nm, expressing the results as mg gallic acid equivalent 100 g ^−1^ fresh weight (mean ± SD) by using a calibration curve previously performed.

### 4.4. Stadistical Analysis

Statistical analysis was determined using the software IBM SPSS Sadistic v. 22.0 for Windows. An analysis of variance (ANOVA) was used to determine the effects of the treatments in the different parameters. Significant differences were identified using Duncan’s test at a significance level of *p* < 0.05.

## 5. Conclusions

This research demonstrates for the first time that the foliar spray treatment with 24-BL is a useful strategy not only for increasing crop yield but also for improving commercial and functional quality of ‘Sunburst’ and ‘Skeena’ sweet cherry. The doses of 0.01 µM and 0.1 µM showed the best effect on the most important quality parameters, 0.1 µM being the only doses capable of significantly increasing commercial crop yield for both cultivars. These results, together with the reduced cracking by 24-BL, would positively affect the income for farmers, who are looking for safe, sustainable and economical tools to improve their crops, regardless the adverse environmental conditions. Additionally, the improvement in the content of bioactive compounds would increase the consumption of sweet cherries by consumers, who are looking for fruit with more functionality and positive effects on their health. Future studies should investigate whether these positive effects would be maintained throughout the storage and lead to an increase in fruit shelf-life, which would reduce wasted fruits and economical losses.

## Data Availability

The original contributions presented in this study are included in the article. Further inquiries can be directed to the corresponding author.
